# Effects of the myeloid cell nuclear differentiation antigen on the proliferation, apoptosis and migration of osteosarcoma cells

**DOI:** 10.3892/ol.2014.1811

**Published:** 2014-01-17

**Authors:** CHENGLIANG SUN, CHUANJU LIU, JUN DONG, DONG LI, WEI LI

**Affiliations:** 1 Department of Orthopaedic Surgery, Provincial Hospital Affiliated to Shandong University, Jinan, Shandong 250021, P.R. China; 2 Department of Orthopaedic Surgery and Cell Biology, New York University School of Medicine, New York, NY 10003, USA

**Keywords:** osteosarcoma, sarcoma osteogenic, myeloid nuclear differentiation antigen, overexpression, proliferation, invasion, apoptosis

## Abstract

Despite improvements over the past two decades, the outcome for patients with advanced osteosarcoma remains poor. Targeted therapies have emerged as promising treatment options for various malignancies. However, effective targeted cancer therapies require the identification of key molecules in the pathogenesis of cancer. The aim of this study was to evaluate the value of the myeloid cell nuclear differentiation antigen (MNDA), a member of the interferon-inducible p200 (IFI-200) family, as a therapeutic target for osteosarcoma by analyzing the baseline expression of MNDA in human osteosarcoma cells and determining the effect of MNDA overexpression on the proliferation and apoptosis profiles and migration/invasion ability in osteosarcoma cells. To this end, MNDA mRNA abundance in wild-type sarcoma osteogenic (Saos-2) cells was analyzed using reverse transcription-polymerase chain reaction, proliferation/apoptosis profiles and migration/invasion capacity in Saos-2 cells overexpressing a green fluorescence protein (GFP)-human MNDA fusion protein. Saos-2 cells found to be overexpressing GFP alone were assessed by 3-(4,5-dimethylthiazol-2-yl-2,5-diphenyltetrazolium bromide (MTT) assay, flow cytometric analysis and Matrigel Transwell migration assay. The results demonstrated that MNDA mRNA was significantly less abundant in wild-type Saos-2 cells compared with human monocyte-like U-937 cells and MNDA overexpression effectively inhibited proliferation, induced apoptosis and reduced migration/invasiveness in Saos-2 cells compared with GFP overexpression alone. Preliminary observations suggested that MNDA potentially serves as a novel therapeutic target for osteosarcoma.

## Introduction

Osteosarcoma is the most common type of primary bone cancer (1). It has been documented that osteosarcoma has a bimodal age-associated pattern of distribution with the first peak occurring during the period of puberty and the second peak at >65 years old (2–4). Although the incidence of osteosarcoma in childhood and adolescence is relatively consistent worldwide (5,6), geographic- and ethnicity-associated variations are evident in the incidence rates of osteosarcoma in other age groups, particularly in the elderly (≥60 years), ranging from 0 cases/million individuals in Kuwait to 11.8 cases/million individuals in the Philippines.

Significant advances have been made recently in diagnostic and palliative local treatments (such as isolated limb perfusion, radiation therapy, embolization, chemoembolization, thermal ablation and cryoablation) of osteosarcoma (7). As a result, the outcome for patients with non-metastatic osteosarcoma has been markedly improved (8). However, patients with a recurrent metastatic disease at diagnosis or with a recurrent disease have a poor prognosis, with the long-term survival rate being only 20% (9). It is therefore crucial to explore innovative strategies in order to effectively manage metastatic and recurrent osteosarcoma.

Therapies targeting key molecules in tumorigenesis have produced exciting and promising developments for cancer treatment in recent years. Recent evidence has indicated that the interferon-inducible p200 (IFI-200) family of proteins may serve as potential therapeutic targets and/or diagnostic biomarkers (10). Consisting of six members (Ifi202a, Ifi202b, Ifi203, Ifi204, Mndal and Aim2) in mice and four homologues, (IFI16, MNDA, IFIX and AIM2) in humans, the IFI-200 family proteins share a high homology each with a conserved domain of 200 amino acids (11,12) and possess antimicrobial, cell growth regulatory, differential regulatory and immunomodulatory properties (13,14). Human and animal studies have demonstrated that the expression of MNDA is downregulated in myelodysplastic syndrome (15), and is markedly (1,000-fold) increased in a mouse strain resistant to B-cell plasmacytoma but completely absent in another mouse strain susceptible to B-cell plasmacytoma (16). The above observations suggest that MNDA is a tumor suppressor. However, the role of MNDA in bone tumors has not been established. The objective of this study was to determine the effect of MNDA overexpression on osteosarcoma cell proliferation and migration/invasiveness in an *in vitro* cell culture system.

## Materials and methods

### Cell culture

An osteosarcoma cell line, sarcoma osteogenic (Saos-2), was purchased from the Shanghai Academy of Life Sciences (Shanghai, China). For maintenance, the cells were cultured in DMEM (Invitrogen, Grand Island, NY, USA) and supplemented with 10% fetal bovine serum (FBS), 1% l-glutamine and 1% penicillin/streptomycin at 37°C in a humidified 5% CO_2_ atmosphere.

### Determination of endogenous MNDA mRNA abundance in wild-type Saos-2 cells

In order to analyze the expression level of endogenous MNDA in Saos-2 cells, MNDA mRNA abundance was determined by reverse transcription-polymerase chain reaction (RT-PCR) with the human monocyte-like U937 cell line included as a positive control. Total RNA was extracted from wild-type Saos-2 cells and U937 cells using TRIzol reagent (Life Technologies, Grand Island, NY, USA). First-strand cDNAs were synthesized with the PrimeScript 1st strand cDNA Synthesis kit from Takara (Tokyo, Japan) according to the manufacturer’s instructions. PCR amplification of the test gene MNDA and the internal control gene 3-glyceraldehyde-phosphate dehydrogenase (GAPDH) were performed with the Premix Taq version 2.0 kit (Takara), using the following reaction conditions: initial denaturation at 95°C for 5 min, followed by 32 main cycles (94°C for 45 sec, 60°C for 45 sec, 72°C for 45 sec), and one final extension cycle at 72°C for 10 min. PCR products were separated by electrophoresis on a 1.5% agarose gel. MNDA and GAPDH bands were visualized by ethidium bromide staining. The MNDA primer sequences used were: sense, 5′-CCACCGCAAGAAACAAAACTGACATCGG-3′ and antisense, 5′-TAAATGGCGCTGTTGCTTTCAGTAC CAC-3′, and the GAPDH primer sequences were: sense, 5′-TGTTGCCATCAATGCCCCTT-3′ and antisense, 5′-CTCC ACGACGTACTCAGCG-3′.

### Expression vector construction

Total cellular RNA was extracted from the U937 cell line (ATCC, Manassas, VA, USA) with TRIzol reagent according to the manufacturer’s instructions. Full-length MNDA cDNA was synthesized by RT and amplified by PCR using a pair of primers designed based upon the human MNDA gene sequence (GenBank accession NM_002432.1) and cloned into pIRES2-enhanced green fluorescent protein (EGFP) (Clontech Laboratories, Inc., Mountain View, CA, USA). Positive pIRES2-EGFP-MNDA clones were selected and confirmed by restriction enzyme mapping and DNA sequencing.

### MTT assay

Saos-2 cells were seeded in triplicate in a 96-well plate at 1×10^3^ cells/well in FBS-containing medium. The next day the cells were transfected with the vehicle (control), pIRES2-EGFP and pIRES2-EGFP-MNDA, respectively, using Lipofectamine 2000 (Life Technologies) according to the manufacturer’s instructions. At 12, 24, 48 and 72 h following transfection, the DNA-lipofectamine-containing medium was removed and 20 μl (5 mg/ml) 3-(4,5-dimethylthiazol-2-yl-2,5-diphenyltetrazolium bromide (MTT) (Sigma-Aldrich, St. Louis, MO, USA) solution was added to each well. Following incubation in the dark for 4 h, the MTT-containing medium was removed and 150 μl DMSO (Sigma-Aldrich) was added. Following 15 min of agitation, the plate was read on an automated plate reader (Perkin-Elmer, Waltham, MA, USA) and the optical density (OD) at 490 nm (OD_490_) was obtained for each well. The percentage growth inhibition in Saos-2 cells transiently transfected with pIRES2-EGFP or pIRES2-EGFP-MNDA was calculated as follows: percentage growth inhibition = (control OD_490_ - test OD_490_)/control OD_490_ × 100. Where the control OD_490_ was the average OD_490_ of the triplicate wells of control Saos-2 cells, while the test OD_490_ was the average OD_490_ of the triplicate wells of Saos-2 cells transfected with pIRES2-EGFP or pIRES2-EGFP-MNDA. Experiments were repeated three times.

### Flow cytometric analysis of apoptosis and cell cycle

Saos-2 cells were seeded in 6-cm dishes at 1×10^5^/well and transfected with the vehicle (control), pIRES2-EGFP and pIRES2-EGFP-MNDA, respectively, as described previously (10). At 48 h following transfection, the transfectants were collected and suspended in PBS in a Falcon^®^ 12 × 75 mm tube. Following washing with PBS, the cells were fixed in ethanol and stained with 50 μg/ml Annexin V-fluorescein isothiocyanate (FITC) (BD Biosciences, San Jose, CA, USA) and 20 μl of 500 μg/ml propidium iodide (PI) (Sigma-Aldrich) at 4°C overnight. Stained cells were analyzed by flow cytometry (Ex, 488 nm; Em, 530 nm). Experiments were performed three times.

### Transwell invasion assay

Saos-2 cells were seeded onto the Matrigel-coated upper chambers (inserts) of a Corning 24-well Transwell plate (Sigma-Aldrich) at 5×10^4^ cells/well and transfected with the vehicle and expression vectors pIRES2-EGFP and pIRES2-EGFP-MNDA in serum-free DMEM, as described previously (15). The lower chambers of the plate were filled with DMEM with 10% FBS as a chemoattractant. After being cultured at 37°C in 5% CO_2_ for 24 h, the insert was carefully removed. Cells that did not migrate through the pores and remained on the upper side of the filter membrane were gently removed with a cotton swab. Cells on the lower side of the filter insert were quickly fixed in 5% glutaraldehyde for 10 min and stained with crystal violet (Sigma-Aldrich). The number of cells on the lower side of the filter insert were counted under a light microscope (Nikon, Melville, NY, USA). Triplicate wells were used for the vehicle transfection and each of the expression vectors respectively. The experiments were each repeated three times.

### Statistical analysis

Data were expressed as the means ± SD and analyzed using the Student’s t-test using statistical analysis software SPSS 12.0 (SPSS Inc., Chicago, IL, USA). P<0.05 was considered to indicate a statistically significant difference.

## Results

### Endogenous MNDA mRNA abundance in wild-type Saos-2 cells

**Figure 1:**
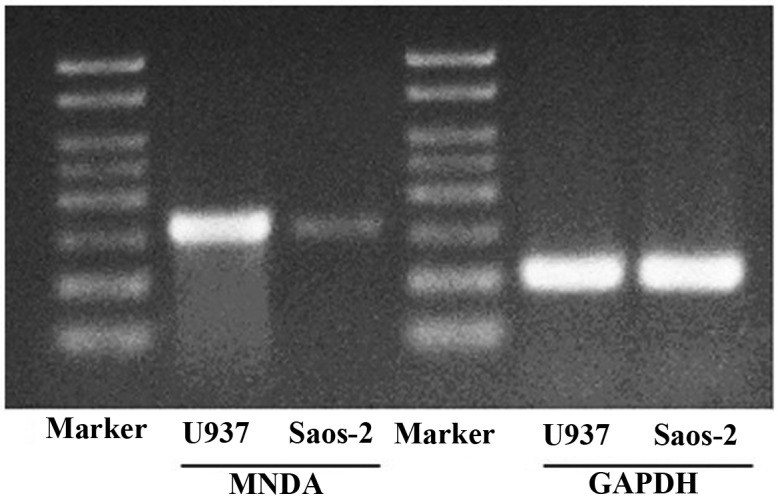
Representative image of ethidium bromide-stained reverse transcription-polymerase chain reaction (RT-PCR) products on an agarose gel, showing myeloid cell nuclear differentiation antigen (MNDA) and 3-glyceraldehyde-phosphate dehydrogenase (GAPDH) bands in U-937 monocyte-like cells and sarcoma osteogenic (Saos-2) cells.

Abundant MNDA mRNA was detected in U937 cells. Compared with the U937 cells, Saos-2 cells had a lower abundance of MNDA mRNA (Fig. 1).

### Effect of MNDA overexpression on the proliferation of osteosarcoma cells

**Figure 2:**
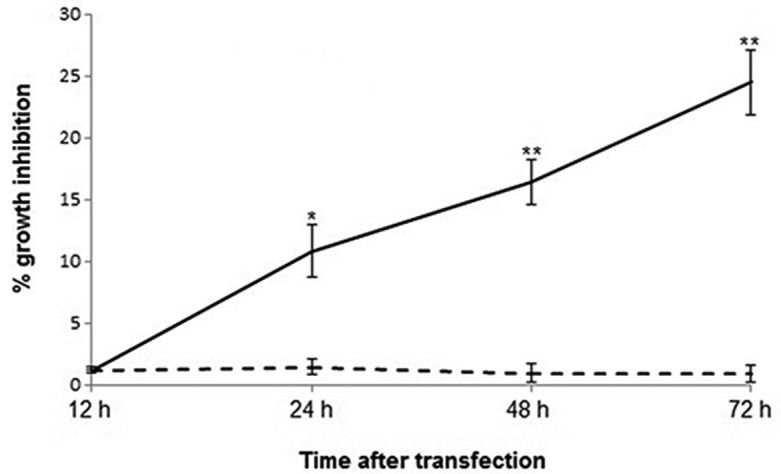
Percentage growth inhibition in sarcoma osteogenic (Saos-2) cells overexpressing pIRES2-enhanced green fluorescent protein (EGFP)-myeloid cell nuclear differentiation antigen (MNDA) and pIRES2-EGFP, respectively, as calculated against wild-type Saos-2 cells at the indicated times. ^*^P<0.05 and ^**^P<0.01, respectively.

In order to determine the effect of MNDA on the proliferation of osteosarcoma cells and cell viability in Saos-2 cells transiently transfected with the vehicle, pIRES2-EGFP-MNDA and pIRES2-EGFP, respectively, cells were assessed by MTT assay. Overexpression of MNDA resulted in a time-dependent increase in the growth inhibition of Saos-2 cells (Fig. 2). At 12 h following transfection, there was no significant difference in cell viability between pIRES2-EGFP-MNDA- and pIRES2-EGFP-transfected Saos-2 cells (P>0.05). However, the percentage growth inhibition calculated against wild-type Saos-2 cells became significant 24 h after transfection. For pIRES2-EGFP-MNDA- and pIRES2-EGFP-overexpressing Saos-2 cells (10.87 vs. 1.51%, P<0.05), the difference became significant 48 h (16.43 vs. 1.01%, P<0.01) and 72 h (24.56 vs. 0.96%, P<0.01) after transfection, respectively.

### Effect of MNDA overexpression on osteosarcoma cell apoptosis

**Figure 3:**
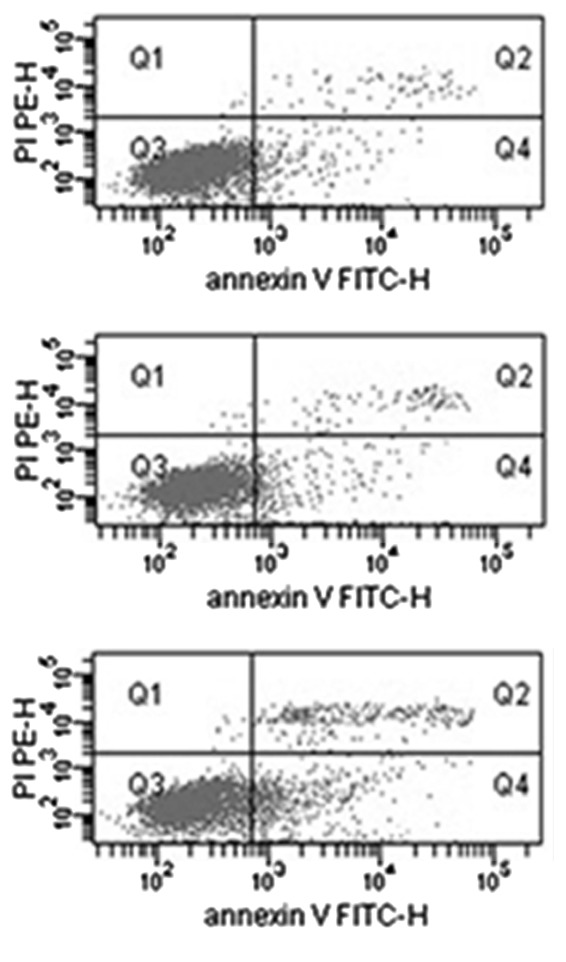
Representative flow cytometric scatter plots of wild-type, pIRES2-enhanced green fluorescent protein (EGFP)-myeloid cell nuclear differentiation antigen (MNDA)-transfected and pIRES2-EGFP-transfected sarcoma osteogenic (Saos-2) cells double-stained with Annexin V-fluorescein isothiocyanate (FITC) and propidium iodide (PI), revealing the number of apoptotic cells.

Fig. 3 shows representative flow cytometric scatter plots of wild-type, pIRES2-EGFP-MNDA- and pIRES2-EGFP-transfected Saos-2 cells double-stained with Annexin V-FITC and PI, showing the number of apoptotic cells 48 h after transfection. When the data from three replicated experiments were analyzed, the average percentage of apoptotic cells was significantly higher (P<0.05) in Saos-2 cells transfected with pIRES2-EGFP-MNDA (17.0%) compared with the wild-type Saos-2 cells (5.7%) and Saos-2 cells transfected with pIRES2-EGFP (5.8%).

### Effect of the MNDA overexpression osteosarcoma cell migration/invasion

**Figure 4:**
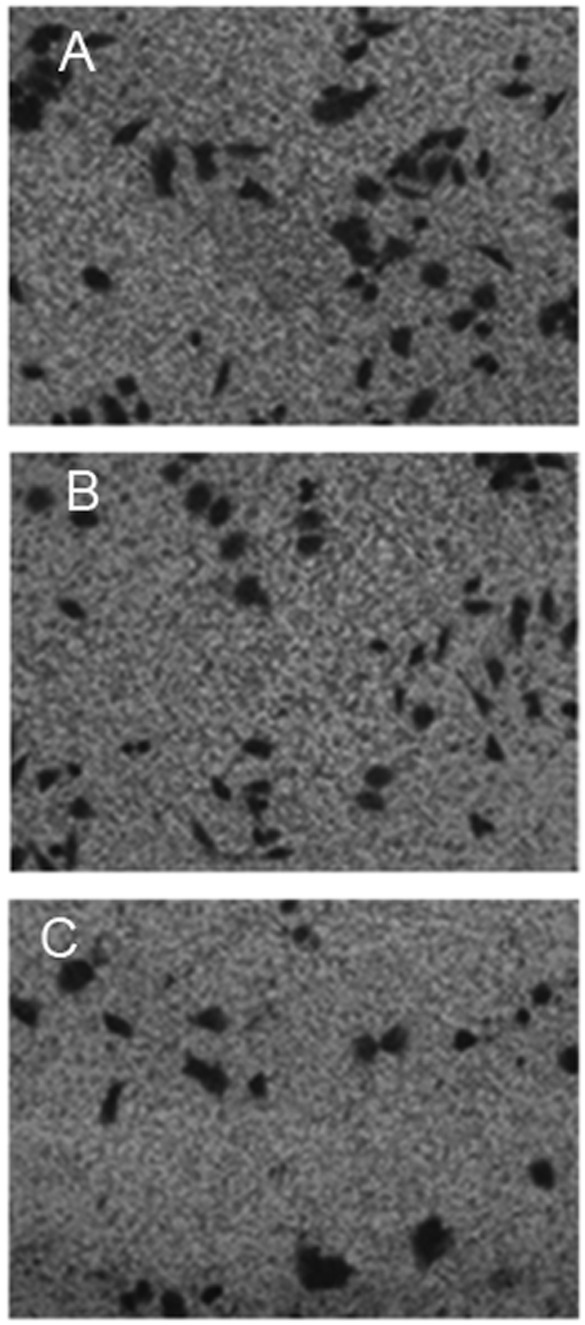
Representative images of (A) wild-type and (B) pIRES2-enhanced green fluorescent protein (EGFP)-myeloid cell nuclear differentiation antigen (MNDA)-overexpressing and (C) pIRES2-EGFP-overexpressing sarcoma osteogenic (Saos-2) cells that migrated through the Matrigel-coated Transwell membrane at 24 h after transfection.

Fig. 4 shows representative images of wild-type Saos-2 cells, pIRES2-EGFP-MNDA- and pIRES2-EGFP-transfected Saos-2 cells that migrated out of the Transwell membrane through the Matrigel matrix. When the average over the three experiments was calculated, the number of pIRES2-EGFP-MNDA-overexpressing Saos-2 cells that migrated through the Matrigel and Transwell membrane was 28±3, which was significantly lower (P<0.01) than that of the wild-type control cells (77±5) and cells overexpressing pIRES2-EGFP (80±3).

## Discussion

Targeted cancer therapies, also known as ‘molecularly targeted drugs’ or ‘molecularly targeted therapies’, have been used for various malignancies in a clinical setting (17). Since interfering with specific molecules involved in tumorigenesis and the subsequent blocking of tumor growth and progression are the fundamental bases of targeted cancer therapies, the identification of appropriate targets (molecules known to play a key role in cancer cell growth and survival) is the key initial step in the development of targeted therapies.

MNDA, a member of the human IFI-200 family, may serve as a novel molecule for targeted therapies. Although MNDA was originally identified in, and thought to be restricted to, myeloid cells (11) there is evidence that MNDA and other IFI-200 family proteins are also expressed in a variety of non-myeloid cell types (18). Somatic inactivation of the genes of the IFI-200 family have been observed in several solid tumors including prostate cancer, melanoma and colon cancer (19–21), suggesting a tumor suppressor role for these family gene products. In a previous study, we demonstrated that MNDA was highly expressed in normal bone tissue but was expressed at markedly low levels in osteosarcoma cells (22). In this study, we observed that MNDA mRNA was significantly less abundant in Saos-2 cells compared to U937 cells (Fig. 1), confirmation of the downregulation of MNDA expression in human osteosarcoma.

In order to evaluate the tumor suppressor role of MNDA in osteosarcoma and explore the possibility of developing an MNDA-based targeted therapy for bone cancers, we also determined the effect of MNDA overexpression on the proliferation, apoptosis and migration/invasion of osteosarcoma cells. Our results demonstrated that the overexpression of MNDA affected osteosarcoma cells by effectively inhibiting proliferation (Fig. 2), inducing apoptosis (Fig. 3) and reducing their migration (Fig. 4) *in vitro*. To the best of our knowledge, we are the first group to have observed MNDA overexpression-mediated cell growth inhibition, cell cycle arrest/apoptosis and reduced invasiveness in osteosarcoma cells. However, the overexpression of other IFI-200 family members has been previously reported to inhibit growth and induce cell cycle arrest, apoptosis or senescence in several other cell models (11,23).

At present, the mechanisms underlying MNDA mediation of growth inhibition, apoptosis and a reduction in the invasiveness of osteosarcoma cells are not completely understood. The majority of IFI-200 family proteins possess a conserved interferon response motif that is associated with protein-protein interactions in the regulation of apoptotic and inflammatory signaling pathways. Furthermore, IFI-200 family proteins may also regulate cell cycle progression/apoptosis and differentiation through interacting with, and modulating the activities of, multiple transcriptional factors such as Rb and p53 (11). Nevertheless, whether similar mechanisms are involved in MNDA-mediated osteosarcoma cell growth inhibition, apoptosis and reduced metastasis requires further investigation.

In conclusion, the present study further confirmed the downregulation of MNDA in osteosarcoma cells as demonstrated in our previous study and has demonstrated that the overexpression of MNDA in osteosarcoma cells results in growth inhibition, cell cycle arrest, apoptosis and reduced invasiveness. These observations suggest that MNDA is a potential novel therapeutic target for osteosarcoma.
